# Counterfactual Explanation-Based Cryptocurrency Price Prediction

**DOI:** 10.3390/e28010065

**Published:** 2026-01-05

**Authors:** Xinxin Luo, Wei Yin

**Affiliations:** 1 School of Cyber Science and Engineering, Southeast University, No.2, Southeast University Road, Jiangning District, Nanjing 211189, China; 2 School of Economics and Management, Southeast University, No.2, Southeast University Road, Jiangning District, Nanjing 211189, China

**Keywords:** counterfactual explanation, time series prediction, cryptocurrency

## Abstract

While deep learning models have demonstrated superior performance in cryptocurrency forecasting, their deployment is often hindered by a lack of interpretability and trustworthiness. To bridge this gap, this paper proposes the Cryptocurrency Counterfactual Explanation (CryptoForecastCF) model. Recognizing the inherent volatility and complex non-linear dynamics of cryptocurrency markets, we argue that understanding the sensitivity of model outputs to slight variations in historical conditions is fundamental to robust risk management. CryptoForecastCF employs a gradient-based optimization strategy to generate meaningful counterfactual explanations. Specifically, it identifies minimal modifications, defined as the optimal perturbations to historical market features such as price constrained by $\ell_{1}$ or $\ell_{2}$ norms, that are sufficient to steer the model’s future predictions into user-specified target intervals. This approach not only elucidates the key driving factors and decision boundaries of opaque models but also equips traders and risk managers with actionable insights, enabling them to identify the specific market shifts required to navigate high-stakes scenarios and mitigate unfavorable predictive outcomes.

## 1. Introduction

The cryptocurrency market constitutes a highly dynamic financial ecosystem characterized by pronounced stochastic volatility, complex non-linear dynamics, and heterogeneous information-driven behaviors [1,2]. Its price trajectories and risk profiles are governed by an intricate interplay of internal market mechanisms, such as order book depth and trading volume, and on-chain activities, while remaining acutely sensitive to multidimensional external factors, including macroeconomic indicators, regulatory shifts, and social media sentiment [3].

To navigate this complexity, deep learning architectures have emerged as the dominant paradigm, evolving from fundamental recurrent units to sophisticated hybrid frameworks that significantly outperform traditional statistical models in forecasting tasks [4,5,6,7]. Early approaches, such as the Long Short-Term Memory (LSTM) models utilized by Hoa et al. [8], demonstrated effectiveness in regression tasks, specifically for closing price prediction using daily OHLCV data. To capture more intricate local and temporal patterns, subsequent research has pivoted toward hybrid architectures. For instance, Amirshahi et al. [9] integrated Convolutional Neural Networks (CNNs) with LSTMs and Multi-Layer Perceptrons (MLPs) to classify high-frequency trends. Similarly, mixed recurrent architectures combining LSTM and Gated Recurrent Unit (GRU) modules, as proposed by Kaur et al. [10], have proven capable of handling multi-time window predictions and modeling complex interdependencies. Furthermore, the incorporation of attention mechanisms into CNN-LSTM structures, as demonstrated by Peng et al. [11], has significantly enhanced the capacity to process multi-frequency and multi-currency data for robust trend forecasting. By automatically extracting latent feature patterns from high-dimensional data, these models effectively integrate information across related assets, thereby enhancing both modeling efficiency and predictive accuracy [12].

However, the superior performance of these deep models comes at the cost of transparency. Their inherent opacity, resulting from complex internal non-linear mappings, renders decision-making processes inaccessible to human reasoning, posing severe challenges to interpretability [13]. In the high-stakes domain of cryptocurrency trading and risk management, this “black-box” nature directly undermines trustworthiness [14,15]. Stakeholders, ranging from institutional investors to regulatory authorities, require more than accurate point predictions; they urgently need to comprehend the key drivers behind model outputs and the boundary conditions under which models may fail. Trust, built upon deep understanding, is a prerequisite for integrating these predictive tools into decision-making workflows.

To enhance model transparency, the research community has developed various post-hoc explanation methods, including gradient-based saliency maps [16], local surrogate models like LIME, and game theoretic approaches such as SHAP. While these methods effectively quantify feature importance [17], they primarily address the question of “attribution”, explaining why a model made a specific prediction in the past. Crucially, they lack “actionability,” failing to provide guidance on how to intervene to alter an unfavorable outcome. For instance, when a model forecasts a high-risk market state, users derive limited utility from a static heatmap of feature importance; rather, they require identification of the minimal, realistic modifications to historical conditions that would have averted such a prediction.

Counterfactual Explanations [18,19] offer a promising avenue to address this limitation by providing actionable recourse. The core objective of a counterfactual explanation is to answer “what-if” questions: what is the minimal, constraint-compliant perturbation to the original input that shifts the model’s output to a predefined target state? This paradigm has been successfully applied in image recognition [20] and natural language processing [21] and is beginning to be explored in time series classification [22]. Its primary advantage lies in its model-agnostic nature and the provision of intuitive recommendations that reveal model sensitivities and decision boundaries [23,24].

Despite these advancements, a significant gap remains in applying counterfactuals to financial time series. As categorized in the comprehensive survey by Guidotti et al. [25], existing methods predominantly rely on generic optimization strategies [26,27] or heuristic search algorithms [28] tailored for static tabular data. Although recent works such as ForecastCF [29] and CounTS [30] have extended these concepts to the time-series domain, they function primarily as general-purpose tools. Consequently, they fail to address the unique challenges inherent to the cryptocurrency market, specifically its extreme volatility and the critical requirement for actionable, interval-based risk thresholds rather than precise point targets.

To bridge this gap, inspired by ForecastCF [29], this study proposes CryptoForecastCF, a novel counterfactual explanation framework specifically designed for cryptocurrency risk prediction. Our work distinguishes itself from existing approaches through three key dimensions. First, unlike standard optimization methods (e.g., WACH [26]) that target precise point-wise decision flips, CryptoForecastCF introduces a novel interval-based optimization objective. This aligns with practical trading needs such as steering predictions into a safe “non-liquidation” range rather than achieving an arbitrary specific value. Second, in contrast to methods prioritizing explanation diversity (e.g., DiCE [27]), we prioritize economic feasibility. By enforcing strict $\ell_{1}/\ell_{2}$ norm constraints, we ensure that generated counterfactuals represent minimal, realistic market shifts rather than theoretical statistical artifacts. Finally, unlike self-interpretable frameworks like CounTS [30] that require specific Bayesian architectures, our approach is model-agnostic and post-hoc. This allows it to provide interpretability for the wide array of pre-trained, high-performance deep learning models currently deployed in quantitative finance.

**Figure 1:**
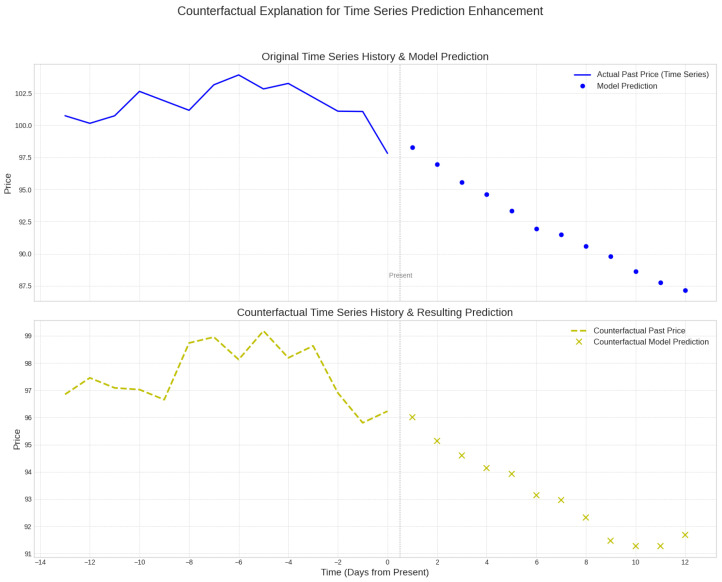
Cryptocurrency Counterfactual Example. The framework generates a modified historical sequence (counterfactual) that shifts the future prediction into a user-defined safe interval.

As illustrated in Figure 1, we envision a scenario where counterfactual explanations enhance the trustworthiness of volatility predictions. Suppose a model predicts an unfavorable 12-day price trend based on a 14-day history. The counterfactual explanation method generates a modified historical price sequence that is very close to the original history but contains targeted perturbations. When the prediction model uses this counterfactual history as input, its generated prediction results successfully fall within the user-predefined desired target interval. This provides insights beyond simple attribution, clearly indicating which temporal dynamics were pivotal in driving the unfavorable forecast.

Based on this analysis, this research addresses the core question: How can we design effective counterfactual explanation methods for cryptocurrency prediction models that provide actionable interpretability guidance? We focus on three sub-problems: (1) defining a counterfactual framework suitable for high-dimensional, multivariate cryptocurrency data; (2) designing generation algorithms that guide predictions toward specific desired intervals; and (3) ensuring the economic feasibility of the recommended interventions. The core contributions of this paper are summarized as follows:

- Problem Formalization: We systematically define the problem of counterfactual explanations for cryptocurrency prediction, demonstrating its critical value in enhancing model transparency and providing actionable decision support in high-risk financial environments.
- The CryptoForecastCF Framework: We propose a universal, gradient-optimization-based framework capable of generating counterfactuals for complex black-box models. It uniquely incorporates interval-based constraints (upper and lower bounds) to align with practical risk management strategies.
- Empirical Evaluation: We conduct extensive experiments on representative cryptocurrency datasets across multiple mainstream deep learning architectures, systematically validating the capability of CryptoForecastCF to generate effective, meaningful, and minimally modified counterfactual explanations.

## 2. Problem Definition

The inherent volatility and non-linear complexity of cryptocurrency markets render accurate forecasting a non-trivial task. While deep learning models have achieved superior performance, their “black-box” nature often obscures the decision-making process, limiting their trustworthiness in high-stakes financial applications. To bridge the gap between predictive performance and interpretability, this study addresses a fundamental question: How can we generate counterfactual explanations that elucidate the specific conditions under which a model yields a desired prediction outcome?

Formally, the objective is to identify minimal yet plausible perturbations to the historical input data such that the model’s output, over a specified future horizon, conforms to user-defined interval constraints. These counterfactual explanations not only enhance model transparency but also provide actionable insights for risk management and strategic investment.

### 2.1. Mathematical Formulation

Let $X=\langle x_{1},x_{2},\ldots ,x_{n}\rangle$ denote a multivariate time series, where $x_{t}\in R^{F}$ represents an *F*-dimensional feature vector at time step *t*. Consider a pre-trained predictive model $f:R^{d\times F}\to R^{T}$, which maps a historical lookback window of length *d* to a prediction sequence of length *T*. The prediction process at time *n* is defined as:

(1) $${\hat{Y}}_{n+1:n+T}=f\left(X_{n-d+1:n}\right)=\langle {\hat{y}}_{n+1},{\hat{y}}_{n+2},\ldots ,{\hat{y}}_{n+T}\rangle$$

where $X_{n-d+1:n}\in R^{d\times F}$ denotes the input matrix from the lookback window, and ${\hat{y}}_{i}\in R$ is the predicted value at future time step *i*.

To incorporate user intent, we define lower and upper bound constraint vectors, $\alpha ,\beta \in R^{T}$, which delineate the acceptable range for the prediction trajectory. These bounds can be dynamically generated via functions $\alpha =\gamma_{lb}\left(X,T\right)$ and $\beta =\gamma_{ub}\left(X,T\right)$, instantiated based on statistical properties, technical indicators (e.g., support/resistance levels), or risk thresholds.

### 2.2. Problem Statement

Based on the aforementioned notation, we formally define the Interval-based Cryptocurrency Counterfactual Prediction problem.

**Definition** **1** (Counterfactual Generation). *Given a time series* X*, a predictive model f, and target constraints* {α,β}*, the goal is to synthesize a counterfactual input sequence* $X^{\prime }$ *that satisfies the following criteria:*

- *Locality: Perturbations are strictly confined to the lookback window, i.e.,* $X_{1:n-d}^{\prime }=X_{1:n-d}$*, ensuring historical integrity outside the relevant context.*
- *Validity: The modified input generates a valid counterfactual prediction* ${\hat{Y}}_{n+1:n+T}^{\prime }=f\left(X_{n-d+1:n}^{\prime }\right)$*.*
- *Constraint Adherence: The counterfactual prediction must lie within the target interval for all* j∈[1,T]*, such that* $\alpha_{j}\leq {\hat{y}}_{n+j}^{\prime }\leq \beta_{j}$*.*
- *Minimality: The counterfactual input* $X_{n-d+1:n}^{\prime }$ *must remain proximal to the original input* $X_{n-d+1:n}$ *to ensure plausibility. This is achieved by minimizing a distance metric* $\mathrm{dist}(X_{n-d+1:n}^{\prime },X_{n-d+1:n})$*.*

We formulate this task as a regularized optimization problem. To facilitate gradient-based solutions, we express the objective function as:

(2) $$X_{n-d+1:n}^{\prime }=\underset{X^{*}\in P}{\arg\min}\left(L_{bounds}\left(f\left(X^{*}\right),\alpha ,\beta \right)+\lambda \cdot \mathrm{dist}\left(X^{*},X_{n-d+1:n}\right)\right)$$

where $X^{*}$ represents the candidate input, $L_{bounds}$ is a penalty function enforcing interval constraints, λ>0 is a regularization coefficient balancing validity and proximity, and P denotes the feasible domain of the input space.

## 3. Proposed Method: CryptoForecastCF

To address the interpretability deficit in cryptocurrency forecasting, we propose CryptoForecastCF, a gradient-based counterfactual generation framework. In contrast to conventional attribution methods that are limited to identifying feature importance, CryptoForecastCF reframes the explanation task as a constrained optimization problem within the input feature space. The framework is engineered to synthesize counterfactual inputs that satisfy three critical criteria: (1) validity, ensuring the model’s predictive output shifts into a user-specified target interval; (2) proximity, minimizing deviation from the original data manifold to preserve realism; and (3) actionability, providing feasible guidance for risk mitigation.

### 3.1. Objective Function Formulation

The optimization objective involves iteratively perturbing the historical lookback window $X_{n-d+1:n}$ to satisfy predefined prediction constraints. A composite objective function L is defined to balance the trade-off between constraint satisfaction and perturbation minimization:

(3) $$L\left(X^{*}\right)=L_{bounds}\left(f\left(X^{*}\right),\alpha ,\beta \right)+\lambda \cdot L_{dist}\left(X^{*},X_{n-d+1:n}\right)$$

where $X^{*}$ represents the candidate counterfactual input, and λ is a regularization hyperparameter governing the strength of the penalty term.

#### 3.1.1. Boundary Constraint Loss

To constrain the prediction output ${\hat{Y}}^{*}=f\left(X^{*}\right)$ within the target interval [α,β], a rectified boundary loss is employed. This loss function penalizes predictions exclusively when they violate the specified upper or lower bounds:

(4) $$L_{bounds}\left({\hat{Y}}^{*},\alpha ,\beta \right)=\sum_{j=1}^{T}\left(\mathrm{ReLU}\left(\alpha_{j}-{\hat{y}}_{n+j}^{*}\right)+\mathrm{ReLU}\left({\hat{y}}_{n+j}^{*}-\beta_{j}\right)\right)$$

where ReLU(z)=max(0,z). This formulation effectively functions as a dynamic mask, contributing to the gradient calculation only at time steps *j* where the prediction ${\hat{y}}_{n+j}^{*}$ falls outside the feasible region $\left[\alpha_{j},\beta_{j}\right]$. Consequently, the optimization process is directed solely toward correcting violated constraints, thereby enhancing convergence efficiency.

#### 3.1.2. Proximity Regularization

To ensure that the generated counterfactuals adhere to the principle of minimal change and remain within the data manifold, a distance penalty term $L_{dist}$ is introduced. Two norm-based regularization strategies are provided to tailor the properties of the perturbation:

- $L_{2}$ Norm (Euclidean Distance): $L_{dist}={∥X^{*}-X_{n-d+1:n}∥}_{2}^{2}$. This formulation encourages smooth, dense perturbations distributed across multiple features, minimizing the aggregate magnitude of change.
- $L_{1}$ Norm (Manhattan Distance): $L_{dist}={∥X^{*}-X_{n-d+1:n}∥}_{1}$. This formulation promotes sparsity, modifying only a subset of critical features, which facilitates the generation of more interpretable and concise explanations.

### 3.2. Optimization Strategy

Given that the predictive model *f* is differentiable (e.g., a deep neural network), gradient descent is utilized to minimize Equation (3). The optimization process updates the input $X^{*}$ while holding the model parameters θ constant. The update rule at iteration *k* is defined as:

(5) $$X_{k+1}^{*}\leftarrow X_{k}^{*}-\eta \cdot \nabla_{X^{*}}L\left(X_{k}^{*}\right)$$

where η denotes the learning rate. The gradient $\nabla_{X^{*}}L$ is computed via backpropagation through the model *f* to the input layer. The iterative procedure terminates when either the boundary constraints are fully satisfied (i.e., $L_{bounds}=0$) or upon reaching a maximum iteration count. This end-to-end optimization renders CryptoForecastCF model-agnostic, making it applicable to any differentiable architecture, including RNNs, CNNs, and Transformers.

### 3.3. Constraint Instantiation Protocols

The definition of semantically meaningful target boundaries α and β is critical for generating actionable explanations. Four instantiation protocols are proposed to address diverse analytical objectives:

- Statistical Trend Constraints: Designed to explore scenarios where predictions deviate from historical norms. Boundaries are derived from the statistics of the lookback window (mean μ, standard deviation σ):
  (6) $$\begin{array}{cc} \alpha_{1} & =\mu \cdot \left(1+s-k\cdot \sigma \right),\;\beta_{1}=\mu \cdot \left(1+s+k\cdot \sigma \right) \end{array}$$
  (7) $$\begin{array}{cc} \alpha_{T} & =\alpha_{1}+\mu \cdot \delta ,\;\beta_{T}=\beta_{1}+\mu \cdot \delta \end{array}$$
  where *s* is a shift factor, *k* controls the interval width, and δ represents the expected trend slope. Intermediate bounds are generated via interpolation.
- Technical Analysis Levels: Incorporates domain knowledge by setting boundaries based on key support and resistance levels, enabling traders to simulate “breakout” or “rebound” scenarios.
- Risk Thresholds: For risk management, β is set to a maximum acceptable Value-at-Risk (VaR) or volatility index, identifying conditions that would trigger risk alerts.
- ROI Objectives: Tailored for quantitative trading, where boundaries define a minimum return on investment (ROI) or maximum drawdown, facilitating strategy backtesting and stress testing.

## 4. Experimental Evaluation

### 4.1. Data Preparation

To rigorously evaluate the efficacy of the proposed CryptoForecastCF framework, this study utilizes a comprehensive suite of cryptocurrency time series datasets. The primary data sources include daily 18 cryptocurrencies, acquired from major exchanges (e.g., Binance, Coinbase) via robust data aggregators such as CoinMarketCap and CryptoCompare. The temporal scope of the dataset extends from 22 September 2020 to 12 March 2024, comprising approximately 1263 daily observations based on closing prices. The dataset incorporates Open, High, Low, Close, and Volume (OHLCV) features, thereby adequately capturing the fundamental stochastic dynamics of the cryptocurrency market.

The hyperparameters for the lookback window *d* and the prediction horizon *T* were established based on standard quantitative trading analysis cycles. Specifically, for daily resolution scenarios, the parameters were set to d=30 (representing a 30-day historical context) and T=7 (forecasting the subsequent weekly trajectory). This configuration strikes an optimal balance between providing sufficient historical context for feature extraction and maintaining the temporal relevance necessary for actionable trading decisions. Data preprocessing adhered to rigorous protocols for time series forecasting. To preserve temporal continuity and ensure a realistic evaluation environment, the dataset was chronologically partitioned into training (70%), validation (15%), and test (15%) subsets. Furthermore, Min-Max normalization was applied to all features to mitigate scale discrepancies and facilitate stable model convergence. In the experimental formulation, $X_{test}$ denotes the original test set samples, while $X_{test}^{\prime }$ represents the generated counterfactual samples, serving as the basis for the subsequent quantitative performance assessment.

The 18 cryptocurrencies selected for this study (detailed in Table 1) constitute a representative cross-section of the digital asset ecosystem, encompassing market leaders (BTC, ETH), exchange utility tokens (BNB), DeFi governance tokens (UNI, LINK), stablecoins (USDT), and prominent altcoins (SOL, ADA, DOT, AVAX). This diverse portfolio is designed to reflect the complex internal correlations and systemic risks inherent in the market. For instance, BTC functions as the market bellwether, exerting significant influence over broader asset classes; ETH, as the premier smart contract platform, serves as a proxy for the utility of the decentralized ecosystem; while USDT liquidity dynamics are critical indicators of overall capital flow.

The descriptive statistics presented in Table 2 reveal significant heterogeneity in the risk return profiles across these assets. First, dominant assets such as BTC and ETH are characterized by high valuations and substantial volatility. BTC’s negative kurtosis (−0.66) suggests a platykurtic distribution with a broad fluctuation range, whereas ETH approximates a normal distribution. Second, assets like BCH and SOL demonstrate elevated relative volatility; notably, BCH’s high kurtosis (3.88) indicates a leptokurtic distribution, implying significant tail risk. Third, the majority of non-stablecoins (e.g., DOGE, AVAX, LTC, ADA) exhibit distinct positive skewness, suggesting a right-tailed distribution likely driven by speculative trading behavior. DOGE is particularly notable for its extreme skewness (1.86) and kurtosis (4.69), reflecting acute sensitivity to market sentiment. Finally, while USDT confirms its stability with a mean approximating 1.0, its extreme kurtosis (67.52) and skewness (5.11) underscore rare but critical de-pegging risks. In summary, the market is characterized by high volatility in large-cap assets, fat tails in altcoins, and tail risks in stablecoins, providing a robust and challenging data foundation for time-series modeling and risk management.

### 4.2. Prediction Models

The CryptoForecastCF framework was evaluated across a diverse set of deep learning architectures, representing the classic method in time series forecasting. To guarantee a rigorous comparative analysis, model-specific architectural hyperparameters were initialized according to standard configurations found in the literature [31,32], while common training hyperparameters were optimized via grid search on the validation set. The selected architectures include:

- **GRU**: As a quintessential Recurrent Neural Network (RNN), the Gated Recurrent Unit excels at capturing long-term temporal dependencies. The optimal configuration identified consists of two stacked GRU layers, each comprising 100 hidden units.
- **Seq2seq [33]**: Leveraging an encoder–decoder framework, this model is adept at handling variable-length sequences for multi-step forecasting. The optimized architecture employs RNN blocks coupled with fully connected layers of 256 units.
- **WaveNet [34]**: This architecture utilizes causal dilated convolutions to capture patterns across varying temporal scales. Superior performance was achieved using a WaveNet structure with residual skip connections and a filter size of 256.
- **N-Beats** [31]: A pure deep neural architecture based on backward and forward residual links and a very deep stack of fully-connected layers. We employed a doubly residual stacked architecture with 256 hidden units.

The lookback window *d* was fixed as a multiple of the prediction horizon *T* (specifically d=30, T=7), adhering to established practices [32]. All models were trained using the Adam optimizer. Hyperparameter tuning on the validation set yielded an optimal learning rate of $1\times 10^{-4}$, a batch size of 128, and a maximum of 100 training epochs. An early stopping strategy with a patience of 10 epochs was implemented to prevent overfitting. The Mean Absolute Error (MAE) served as the objective function for training.

### 4.3. Counterfactual Method Evaluation

In this study, the counterfactual explanations generated by CryptoForecastCF are benchmarked against two baseline methods. The core hyperparameters for CryptoForecastCF were set to a learning rate of η=0.001 and a maximum iteration count of 100. The regularization coefficient λ was fine-tuned on the validation set to ensure the stability and convergence of the optimization process. The baseline methods include:

- **Input Gradient (Grad)** [35]: A gradient-based attribution method that identifies input features or time steps with the highest sensitivity regarding the prediction output. Although it is not strictly a generative counterfactual approach, it serves as a baseline for assessing model sensitivity.
- **BaseShift** [36]: A heuristic baseline that employs a naive shifting strategy, applying fixed shift factors (e.g., the average expected change) to the entire input lookback window to generate “modified” inputs.

### 4.4. Evaluation Metrics

To rigorously evaluate the performance of the proposed framework, we adopt a two-tiered validation strategy focusing on both predictive fidelity and the quality of the counterfactual explanations.

#### 4.4.1. Predictive Performance Metrics

To assess forecasting accuracy, we employ the symmetric Mean Absolute Percentage Error (sMAPE) and the Mean Absolute Scaled Error (MASE) as primary indicators. To ensure statistical robustness, all reported results represent the mean performance derived from five independent experimental trials across the entire test set.

#### 4.4.2. Counterfactual Evaluation Metrics

The quality of the generated counterfactual explanations is evaluated across three distinct dimensions: (1) Effectiveness, quantifying the extent to which the counterfactual predictions satisfy the desired target constraints; (2) Proximity, measuring the deviation of the counterfactual samples from the original input space to ensure minimal alteration; and (3) Plausibility, assessing the realistic feasibility and statistical alignment of the generated perturbations with the underlying data manifold.

##### Effectiveness Metrics

To quantify effectiveness, we utilize the Validity Ratio and the Stepwise Validity AUC. The Validity Ratio computes the mean proportion of time steps within a generated counterfactual sequence that successfully satisfy the target constraints:

(8) $$\mathrm{Ratio}\left({\hat{X}}^{\prime }\right)=\frac{1}{K}\sum_{k=1}^{K}\left(\frac{1}{T}\sum_{i=1}^{T}\tau_{b}\left({\hat{x_{i}^{\prime }}}^{k},\alpha_{i}^{k},\beta_{i}^{k}\right)\right),\;\forall {\hat{x}}^{k}\in {\hat{X}}^{\prime }$$

where $\tau_{b}\left(\cdot \right)$ denotes an indicator function that returns 1 if the prediction falls within the specified bounds $\left[\alpha_{i},\beta_{i}\right]$, and 0 otherwise.

Complementing this, we introduce the Stepwise Validity AUC to assess the temporal continuity of valid predictions. This metric calculates the Area Under the Curve (AUC) for the function ϕ(t), which represents the proportion of counterfactuals that maintain validity for at least *t* consecutive time steps:

(9) $$\text{Step-AUC}\left({\hat{X}}^{\prime }\right)=\int_{t=0}^{1}\phi \left(t\right)\;dt$$

where ϕ(t) is derived from a cumulative validity check $\tau_{c}$, enforcing the strict condition that a time step is considered valid only if all preceding steps also satisfy the constraints. For both metrics, values approaching 1 denote superior performance.

##### Proximity and Plausibility Metrics

To evaluate the minimal alteration and realistic nature of the counterfactuals, we employ the following metrics: Proximity [22] (the average Euclidean distance between the original and counterfactual samples, where lower values are preferred); Compactness [22] (measuring the sparsity of modifications, where higher values indicate more focused changes).

### 4.5. Experimental Results and Discussion

This section presents a comparative evaluation of the proposed CryptoForecastCF method against the BaseNN and BaseShift baselines across four deep learning architectures. The experiments utilize 18 cryptocurrencies daily closing prices from 22 September 2020 to 12 March 2024. Table 3 summarizes the performance across prediction quality, counterfactual effectiveness, and counterfactual quality. Bold entries denote the optimal performance for each model-metric pair.

#### 4.5.1. Multi-Horizon Results

Table 4 reports the aggregated multi-horizon performance over the 18-cryptocurrency test set for T∈{1,7,15,30}. Two consistent patterns emerge. First, forecasting difficulty increases with horizon: both sMAPE and MASE generally rise as *T* grows, reflecting the compounding uncertainty in longer-range cryptocurrency prediction. Second, counterfactual effectiveness remains high at moderate horizons (e.g., T=7 and T=15), but degrades for specific architectures at long horizons (notably Seq2Seq at T=30), indicating that certain model classes can be substantially harder to steer into the desired interval when the forecast window becomes long.

Notably, the Step-AUC values for the 1-day setting are reported as 0.000 across methods. This is expected because the stepwise validity curve becomes degenerate when T=1 (there is no multi-step continuity to accumulate), so the resulting AUC does not provide meaningful discrimination in the single-step case. Therefore, Step-AUC should be interpreted primarily for multi-step horizons (T≥7) where temporal consistency is non-trivial.

#### 4.5.2. Asset-Level Results

Table 5 provides an asset-level breakdown under the default setting (d=30, T=7). The results highlight strong cross-asset heterogeneity: large-cap assets (e.g., BTC, ETH) typically exhibit higher counterfactual validity under N-BEATS/WaveNet/GRU, while more volatile altcoins (e.g., SOL, AVAX) often show substantially lower validity and Step-AUC, indicating that steering predictions into a target interval can be considerably harder in high-volatility regimes. In addition, the per-asset view makes clear that model choice interacts with asset characteristics: for example, Seq2Seq shows severe instability on some assets (e.g., extreme sMAPE for BTC), suggesting that its learned dynamics may be less robust for certain price processes and horizons in this experimental setup.

#### 4.5.3. Prediction Performance Analysis

**sMAPE Metric Analysis:** The CryptoForecastCF framework exhibits exceptional capability in preserving prediction fidelity across all evaluated architectures. Specifically, the sMAPE on the N-BEATS model was reduced to 9.8%, representing a 40.2% improvement over BaseShift (16.4%) and a 28.5% improvement over BaseNN (13.7%). This substantial reduction underscores the capability of CryptoForecastCF to preserve the fidelity of the underlying predictive model while generating counterfactual samples through end-to-end optimization. Notably, the method not only preserves prediction performance during counterfactual generation but also appears to positively influence the prediction accuracy of the underlying models, likely due to the deep utilization of model internal structures during the optimization process.

**MASE Metric Analysis:** In terms of the Mean Absolute Scaled Error, CryptoForecastCF consistently outperformed the baselines. MASE values for all models were maintained below 0.731, significantly surpassing the naive prediction baseline (MASE = 1.0). For the N-BEATS model, the MASE value of 0.672 represents a 28.7% improvement over BaseShift (0.943). These findings corroborate the efficacy of the gradient-based optimization strategy, which systematically navigates the feature space to avoid the accuracy degradation often associated with the heuristic perturbations employed by traditional methods.

#### 4.5.4. Counterfactual Effectiveness Evaluation

**Validity Metric Analysis:** CryptoForecastCF demonstrated superior performance in counterfactual effectiveness. The validity on the N-BEATS model reached 92.4%, indicating that 92.4% of the generated counterfactual samples successfully satisfied the target constraints. This performance significantly surpasses BaseNN (74.3%) and BaseShift (65.1%), yielding improvements of 24.4% and 41.9%, respectively. These results validate the robustness of the gradient-guided optimization, demonstrating its ability to reliably steer predictions across decision boundaries to meet user-defined objectives.

**Step-AUC Metric Analysis:** The Stepwise Validity analysis reveals that CryptoForecastCF excels in maintaining the temporal continuity of valid predictions. The Step-AUC on the Seq2Seq model reached 83.2%, indicating high target consistency across the entire prediction horizon. With an average improvement exceeding 20% compared to baseline methods, this metric highlights the method’s stability in long-sequence forecasting tasks. The superior Step-AUC scores reflect the efficiency of CryptoForecastCF in progressively achieving targets, underscoring the continuity and coherence of its optimization trajectory.

#### 4.5.5. Counterfactual Quality Analysis

**Proximity Metric Analysis:** Regarding the minimization of input modifications, CryptoForecastCF achieved significant optimization. The Proximity value on the Seq2Seq model was 0.159, indicating that only a 15.9% deviation from the original input was required to achieve the expected targets. This represents a 46.6% reduction in perturbation magnitude compared to BaseShift (29.8%) and a 27.4% reduction compared to BaseNN (21.9%). This advantage in identifying minimal necessary changes aligns with the principle of sparsity in counterfactual explanations, thereby enhancing their interpretability and trustworthiness for end-users.

**Compactness Metric Analysis:** The analysis of change concentration demonstrates that CryptoForecastCF effectively localizes modifications to critical temporal segments. The Compactness value on the N-BEATS model reached 87.1%, implying that 87.1% of the modifications were concentrated in a few key positions. This significantly outperforms BaseShift (68.7%) and BaseNN (75.6%). Such a highly concentrated modification pattern improves the actionability of the counterfactual explanations, enabling users to pinpoint the specific temporal events that most significantly influence the model’s predictive outcome.

### 4.6. Ablation Study Analysis

#### 4.6.1. Sensitivity to Desired Change Magnitude

Table 6 indicate that the optimal values for the desired change parameter (desired_change) are predominantly concentrated within the ±0.1 (i.e., ±10%) interval. Within this range, the majority of models achieve a superior equilibrium between counterfactual effectiveness and quality. For instance, the N-BEATS model attains a Validity of 0.848, Step-AUC of 0.697, Compactness of 0.715, and an optimal Proximity of 0.083 when desired_change is set to 0.1. Similarly, GRU and WaveNet exhibit peak performance at desired_change = −0.1. It is noteworthy that while Proximity and Compactness remain exceptional within the ±0.1 interval, the Seq2Seq model demonstrates diminished Validity (e.g., 0.008), suggesting that insufficient perturbation magnitudes may fail to drive certain architectures to generate effective counterfactuals. Conversely, as the absolute value of desired change increases (|desired_change|≥0.2), Validity and Compactness decline across all models, while Proximity increases significantly. This trend suggests that excessive prediction targets compromise the plausibility of the generated counterfactual explanations by necessitating unrealistic input perturbations.

These findings reflect the inherent trade-offs in counterfactual generation: smaller desired_change values favor proximity and plausibility while maintaining adequate effectiveness, whereas larger values disrupt this balance. N-BEATS, WaveNet, and GRU display distinct performance peaks, indicating sensitivity to this parameter, while Seq2Seq’s consistent underperformance suggests limited applicability for this specific method under low-magnitude changes. Given that prediction quality metrics (sMAPE, MASE) reflect inherent model properties unaffected by post hoc counterfactual generation, the optimal desired_change should be selected to maximize counterfactual effectiveness and quality.

Based on this analysis, we recommend an optimal parameter interval of $\Delta^{\in }\left[-0.1,0.1\right]$ for the CryptoForecastCF method. Within this range, the framework consistently achieves optimal or near-optimal comprehensive performance. From an optimization perspective, this interval provides a balanced constraint that facilitates the search for solutions sufficiently close to the original inputs while satisfying the counterfactual objectives.

#### 4.6.2. Impact of Standard Deviation Fraction

Table 7 elucidates the impact of the standard deviation fraction ($\sigma_{frac}$) parameter on the performance of CryptoForecastCF. This parameter defines the permissible search space for counterfactual generation relative to the intrinsic variability of the data. The experimental results reveal a distinct trade-off between effectiveness and counterfactual quality.

Across all four prediction models, setting $\sigma_{frac}=1.0$ consistently yields the optimal balance for predictive accuracy and validity. For instance, the N-BEATS model achieves its lowest sMAPE (9.8%) and highest Validity (92.4%) at this threshold. This finding suggests that aligning the search space with the natural volatility of the data (i.e., one standard deviation) provides sufficient flexibility for the algorithm to identify feasible counterfactuals that satisfy the target constraints without introducing excessive noise.

However, the data also highlights a divergence in optimization objectives. While $\sigma_{frac}=1.0$ maximizes effectiveness, lower parameter values (0.25) result in superior Proximity and Compactness scores. For example, at $\sigma_{frac}=0.25$, N-BEATS achieves a Proximity of 0.089 and Compactness of 0.934, significantly outperforming the values at $\sigma_{frac}=1.0$ (0.145 and 0.871, respectively). This indicates that tighter constraints force the model to make smaller, more concentrated changes, albeit at the cost of reduced Validity (dropping to 75.6% for N-BEATS). Conversely, increasing $\sigma_{frac}$ beyond 1.0 (to 1.5–2.0) degrades both predictive quality and counterfactual sparsity, as the expanded search space allows for larger, less realistic perturbations.

Consequently, we identify $\sigma_{frac}=1.0$ as the robust default configuration for practical applications where high validity is paramount. It ensures high counterfactual effectiveness (>88% across models) while maintaining acceptable similarity to original inputs. However, for scenarios strictly prioritizing minimal modification, a lower fraction may be preferable, provided the associated reduction in validity is acceptable.

## 5. Conclusions

This paper addresses the critical imperative for interpretability in deep learning-based cryptocurrency forecasting by introducing CryptoForecastCF, a rigorous interval-constrained counterfactual explanation framework. By formalizing the problem through the core principles of input modification, prediction validity, constraint satisfaction, and modification minimization, and leveraging a gradient-based optimization strategy with dynamic masking, the proposed method efficiently generates actionable, high-fidelity explanations. Empirical evaluations substantiate the framework’s superiority, demonstrating a greater than 20% improvement in counterfactual effectiveness and a reduction of over 30% in input perturbations compared to baselines. These technical advancements translate into substantial practical utility for the fintech industry: empowering traders to validate algorithmic signals, facilitating precise stress testing via “what-if” simulations, and providing transparent audit trails for regulatory compliance. While the current reliance on white-box gradient access and structured OHLCV data presents certain limitations regarding proprietary systems and multi-modal market drivers, these constraints delineate clear pathways for future investigation. Prospective research will focus on integrating unstructured sentiment data to enable comprehensive market analysis, extending the architecture to Graph Neural Networks to capture systemic risk contagion, and exploring model-agnostic reinforcement learning approaches to eliminate gradient dependencies. Ultimately, CryptoForecastCF establishes a foundational paradigm for trustworthy, transparent, and actionable AI within the high-stakes domain of financial decision-making.

## Figures and Tables

**Table 1:** Nomenclature of Selected Cryptocurrencies.

Symbol	Name	Symbol	Name	Symbol	Name
BTC	Bitcoin	ETH	Ethereum	BNB	Binance Coin
ADA	Cardano	SOL	Solana	XRP	XRP
DOGE	Dogecoin	LTC	Litecoin	DOT	Polkadot
UNI	Uniswap	LINK	Chainlink	BCH	Bitcoin Cash
MATIC	Polygon	TRX	TRON	AVAX	Avalanche
USDT	Tether	ATOM	Cosmos	XLM	Stellar

**Table 2:** Descriptive Statistics of the Cryptocurrency Portfolio.

Variable	Mean	Std. Dev.	Kurtosis	Skewness	Max	Min
BTC	34,266.93	13,485.45	−0.66	0.42	72,455.60	10,260.00
ETH	2087.08	949.74	0.05	0.59	4780.08	322.90
BNB	301.34	136.96	0.40	0.01	671.86	23.10
ADA	0.76	0.62	1.08	1.32	3.013	0.07744
SOL	54.59	55.16	1.48	1.49	258.63	1.197
XRP	0.61	0.28	1.70	1.32	1.814	0.2144
DOGE	0.11	0.09	4.69	1.86	0.6784	0.00249
LTC	109.87	57.98	2.10	1.43	372.25	43.34
DOT	14.12	11.92	0.39	1.23	53.89	3.63126
UNI	11.43	9.12	0.68	1.32	43.11	1.946
LINK	14.98	9.19	0.55	1.06	50.30	5.08762
BCH	321.17	219.67	3.88	1.62	1499.79	89.68
MATIC	0.91	0.55	0.30	0.46	2.86243	0.01229
TRX	0.07	0.03	0.39	0.44	0.1639	0.02424
AVAX	30.98	27.60	1.31	1.47	134.96	2.90
USDT	1.00024	0.00087	67.52	5.11	1.0142	0.9963
ATOM	14.90	8.94	0.48	1.21	43.64	3.893
XLM	0.19	0.13	1.60	1.42	0.702	0.06794

**Table 3:** Performance Comparison of Counterfactual Explanation Methods under Different Deep Learning Models.

Model	Counterfactual Method	Prediction Quality		Counterfactual Effectiveness		Counterfactual Quality	
		sMAPE (%)	MASE	Validity	Step-AUC	Proximity	Compactness
N-BEATS	BaseNN	13.7	0.821	0.743	0.672	0.234	0.756
	BaseShift	16.4	0.943	0.651	0.598	0.312	0.687
	CryptoForecastCF	**9.8**	**0.672**	**0.924**	**0.847**	**0.145**	**0.871**
WaveNet	BaseNN	15.2	0.856	0.714	0.645	0.251	0.743
	BaseShift	18.9	0.987	0.629	0.567	0.327	0.664
	CryptoForecastCF	**11.3**	**0.718**	**0.896**	**0.812**	**0.168**	**0.834**
Seq2Seq	BaseNN	14.3	0.798	0.756	0.698	0.219	0.781
	BaseShift	17.1	0.924	0.673	0.612	0.298	0.702
	CryptoForecastCF	**10.6**	**0.694**	**0.907**	**0.832**	**0.159**	**0.863**
GRU	BaseNN	15.1	0.834	0.732	0.667	0.242	0.769
	BaseShift	17.8	0.961	0.658	0.591	0.308	0.693
	CryptoForecastCF	**12.1**	**0.731**	**0.883**	**0.798**	**0.172**	**0.847**

*Note:* Bold values indicate best performance for each model. sMAPE: symmetric Mean Absolute Percentage Error (lower is better); MASE: Mean Absolute Scaled Error (lower is better); Validity: proportion of counterfactuals achieving desired targets (higher is better); Step-AUC: Area Under Curve of stepwise validity (higher is better); Proximity: average distance to original input (lower is better); Compactness: concentration of changes (higher is better).

**Table 4:** Multi-horizon performance comparison (aggregated over the 18-cryptocurrency test set). Results are reported for different forecasting horizons T∈{1,7,15,30} days.

Horizon *T*	Method	Prediction Quality		Counterfactual Effectiveness		Counterfactual Quality	
		sMAPE (%)	MASE	Validity	Step-AUC	Proximity	Compactness
1-Day	N-BEATS	12.53	3.39	0.818	0.000	0.041	0.865
	WaveNet	4.43	1.27	0.636	0.000	0.008	0.996
	Seq2Seq	3.21	0.86	0.727	0.000	0.013	0.989
	GRU	6.30	1.54	0.636	0.000	0.020	0.978
7-Day	N-BEATS	12.25	3.08	1.000	0.857	0.002	1.000
	WaveNet	12.02	3.08	0.993	0.839	0.001	0.999
	Seq2Seq	10.11	2.65	0.979	0.846	0.008	0.990
	GRU	14.06	3.69	0.971	0.754	0.011	0.980
15-Day	N-BEATS	14.92	3.90	0.957	0.893	0.053	0.774
	WaveNet	15.05	3.90	0.973	0.860	0.008	0.990
	Seq2Seq	28.03	7.22	0.803	0.723	0.045	0.946
	GRU	16.00	4.37	0.937	0.827	0.033	0.957
30-Day	N-BEATS	18.15	4.79	0.958	0.898	0.089	0.670
	WaveNet	21.12	5.57	0.875	0.620	0.045	0.952
	Seq2Seq	149.18	24.07	0.047	0.004	0.103	0.947
	GRU	23.56	6.43	0.813	0.629	0.086	0.908

**Table 5:** Per-cryptocurrency (asset-level) performance for the default setting (d=30,T=7). The table is organized into two parallel sections with horizontal separators for each asset.

Asset	Method	Pred. Quality		Effectiveness		Quality		Asset	Method	Pred. Quality		Effectiveness		Quality	
		sMAPE	MASE	Val.	S-AUC	Prox.	Comp.			sMAPE	MASE	Val.	S-AUC	Prox.	Comp.
BTC	N-BEATS	15.66	4.04	0.99	0.94	0.017	0.908	UNI	N-BEATS	20.22	4.69	0.09	0.00	0.068	0.877
	WaveNet	16.57	4.29	0.93	0.74	0.019	0.977		WaveNet	21.76	4.91	0.08	0.01	0.086	0.923
	Seq2Seq	155.01	24.37	0.00	0.00	0.103	0.945		Seq2Seq	36.90	7.57	0.04	0.00	0.025	0.992
	GRU	17.95	4.98	0.90	0.77	0.037	0.953		GRU	30.20	6.21	0.06	0.01	0.066	0.956
ETH	N-BEATS	17.47	5.45	0.71	0.13	0.249	0.298	LINK	N-BEATS	19.38	4.00	0.57	0.22	0.093	0.777
	WaveNet	11.25	3.84	0.72	0.49	0.069	0.938		WaveNet	16.46	3.81	0.58	0.41	0.068	0.917
	Seq2Seq	93.65	20.71	0.00	0.00	0.078	0.967		Seq2Seq	36.02	6.63	0.16	0.01	0.063	0.973
	GRU	9.11	3.06	0.77	0.42	0.065	0.935		GRU	13.68	3.27	0.63	0.12	0.057	0.940
BNB	N-BEATS	14.36	4.24	0.72	0.43	0.267	0.328	BCH	N-BEATS	19.06	5.21	0.08	0.00	0.021	0.974
	WaveNet	10.52	3.26	0.78	0.50	0.096	0.913		WaveNet	20.38	5.13	0.02	0.00	0.030	0.968
	Seq2Seq	82.76	16.30	0.00	0.00	0.080	0.964		Seq2Seq	43.20	12.06	0.02	0.00	0.002	1.000
	GRU	8.37	2.57	0.73	0.36	0.095	0.908		GRU	35.33	7.48	0.01	0.00	0.004	0.999
ADA	N-BEATS	23.44	4.29	0.16	0.01	0.075	0.831	MATIC	N-BEATS	25.18	4.50	0.91	0.52	0.171	0.464
	WaveNet	22.25	4.28	0.24	0.05	0.077	0.913		WaveNet	20.53	3.56	0.83	0.57	0.081	0.927
	Seq2Seq	36.26	6.32	0.06	0.00	0.046	0.984		Seq2Seq	49.23	6.12	0.17	0.00	0.086	0.960
	GRU	13.31	2.87	0.31	0.03	0.037	0.960		GRU	15.65	2.68	0.74	0.42	0.049	0.950
SOL	N-BEATS	38.25	12.65	0.12	0.00	0.102	0.827	TRX	N-BEATS	10.08	4.17	0.60	0.30	0.091	0.796
	WaveNet	35.58	12.12	0.08	0.00	0.066	0.932		WaveNet	7.15	3.13	0.66	0.48	0.035	0.965
	Seq2Seq	77.74	17.32	0.03	0.00	0.041	0.990		Seq2Seq	53.82	15.81	0.08	0.00	0.079	0.966
	GRU	39.85	13.05	0.03	0.01	0.066	0.943		GRU	9.17	3.80	0.37	0.01	0.062	0.930
XRP	N-BEATS	14.32	3.37	0.45	0.05	0.105	0.770	AVAX	N-BEATS	46.40	14.02	0.06	0.00	0.122	0.784
	WaveNet	18.66	4.30	0.49	0.29	0.120	0.870		WaveNet	57.80	15.08	0.05	0.00	0.118	0.866
	Seq2Seq	56.40	10.32	0.08	0.00	0.057	0.980		Seq2Seq	67.09	17.06	0.01	0.00	0.021	0.994
	GRU	13.06	3.10	0.47	0.01	0.070	0.934		GRU	56.05	15.52	0.03	0.00	0.105	0.924
DOGE	N-BEATS	40.42	8.11	0.20	0.01	0.145	0.579	USDT	N-BEATS	13.07	4.67	0.08	0.00	0.103	0.835
	WaveNet	23.79	5.39	0.34	0.02	0.096	0.895		WaveNet	10.72	3.74	0.21	0.08	0.069	0.883
	Seq2Seq	60.59	11.03	0.03	0.00	0.040	0.992		Seq2Seq	7.83	2.87	0.02	0.00	0.049	0.982
	GRU	9.94	2.44	0.21	0.00	0.029	0.972		GRU	6.63	2.43	0.09	0.00	0.091	0.923
LTC	N-BEATS	12.10	3.02	0.45	0.04	0.083	0.824	ATOM	N-BEATS	13.12	3.08	0.46	0.04	0.084	0.793
	WaveNet	12.22	3.17	0.66	0.26	0.072	0.927		WaveNet	15.58	3.89	0.42	0.08	0.084	0.910
	Seq2Seq	56.20	10.65	0.06	0.00	0.061	0.978		Seq2Seq	44.41	8.69	0.08	0.00	0.051	0.984
	GRU	10.75	2.69	0.33	0.01	0.064	0.934		GRU	15.55	3.91	0.26	0.02	0.066	0.941
DOT	N-BEATS	26.65	9.93	0.03	0.00	0.097	0.823	XLM	N-BEATS	12.26	3.28	0.33	0.01	0.023	0.977
	WaveNet	21.92	7.83	0.03	0.00	0.046	0.964		WaveNet	15.06	3.92	0.28	0.02	0.046	0.954
	Seq2Seq	42.12	17.00	0.00	0.00	0.008	0.999		Seq2Seq	10.58	2.85	0.23	0.02	0.027	0.970
	GRU	28.45	10.04	0.01	0.00	0.073	0.953		GRU	10.74	2.90	0.38	0.00	0.022	0.979

**Table 6:** Ablation Study Results of Desired Change Parameter on CryptoForecastCF Method Performance.

Model	Δ	Pred. Qual.		Effectiveness		Quality		Model	Δ	Pred. Qual.		Effectiveness		Quality	
		sMAPE	MASE	Val.	S-AUC	Prox.	Comp.			sMAPE	MASE	Val.	S-AUC	Prox.	Comp.
N-BEATS	−0.5	12.0	2.87	0.553	0.417	0.208	0.277	Seq2Seq	−0.5	161.8	23.7	**0.136**	**0.004**	**0.067**	**0.953**
	−0.4	12.0	2.87	0.629	0.451	0.171	0.408		−0.4	161.8	23.7	0.083	0.004	0.067	0.952
	−0.3	12.0	2.87	0.674	0.534	0.162	0.425		−0.3	161.8	23.7	0.053	0.004	0.068	0.952
	−0.2	12.0	2.87	0.705	0.545	0.116	0.583		−0.2	161.8	23.7	0.038	0.004	0.069	0.951
	−0.1	12.0	2.87	0.795	0.625	0.084	0.655		−0.1	161.8	23.7	0.008	0.004	0.069	0.950
	0.1	12.0	2.87	**0.848**	**0.697**	**0.083**	**0.715**		0.1	161.8	23.7	0.008	0.004	0.070	0.949
	0.2	12.0	2.87	0.780	0.640	0.105	0.623		0.2	161.8	23.7	0.008	0.004	0.071	0.948
	0.3	12.0	2.87	0.697	0.530	0.158	0.436		0.3	161.8	23.7	0.008	0.004	0.071	0.947
	0.4	12.0	2.87	0.621	0.428	0.176	0.392		0.4	161.8	23.7	0.008	0.004	0.072	0.947
	0.5	12.0	2.87	0.530	0.386	0.208	0.283		0.5	161.8	23.7	0.008	0.004	0.072	0.946
WaveNet	−0.5	9.6	2.34	0.538	0.496	0.043	0.961	GRU	−0.5	12.3	3.21	0.606	0.538	0.065	0.911
	−0.4	9.6	2.34	0.576	0.530	0.033	0.967		−0.4	12.3	3.21	0.636	0.545	0.062	0.914
	−0.3	9.6	2.34	0.614	0.564	0.028	0.970		−0.3	12.3	3.21	0.712	0.598	0.037	0.946
	−0.2	9.6	2.34	0.629	0.580	0.025	0.972		−0.2	12.3	3.21	0.727	0.606	0.036	0.948
	−0.1	9.6	2.34	0.659	**0.583**	**0.023**	**0.973**		−0.1	12.3	3.21	**0.780**	**0.655**	**0.028**	**0.959**
	0.1	9.6	2.34	**0.697**	0.527	0.034	0.968		0.1	12.3	3.21	0.742	0.640	0.036	0.946
	0.2	9.6	2.34	0.621	0.413	0.053	0.961		0.2	12.3	3.21	0.682	0.458	0.076	0.898
	0.3	9.6	2.34	0.561	0.386	0.061	0.955		0.3	12.3	3.21	0.576	0.348	0.090	0.880
	0.4	9.6	2.34	0.492	0.326	0.066	0.949		0.4	12.3	3.21	0.515	0.311	0.091	0.879
	0.5	9.6	2.34	0.447	0.277	0.076	0.936		0.5	12.3	3.21	0.462	0.288	0.088	0.880

Note: Δ denotes the desired change as a percentage relative to the original prediction values. Negative values indicate an expected decrease in prediction, while positive values indicate an expected increase. Pred. Qual. = Prediction Quality; Val. = Validity; Prox. = Proximity; Comp. = Compactness. Bold values indicate the optimal performance for each metric (lower is better for sMAPE, MASE, and Proximity; higher is better for Validity, Step-AUC, and Compactness).

**Table 7:** Ablation Study Results of Standard Deviation Fraction Parameter on CryptoForecastCF Method Performance.

Model	$\sigma_{\mathrm{frac}}$	Pred. Qual.		Effectiveness		Quality		Model	$\sigma_{\mathrm{frac}}$	Pred. Qual.		Effectiveness		Quality	
		sMAPE	MASE	Val.	S-AUC	Prox.	Comp.			sMAPE	MASE	Val.	S-AUC	Prox.	Comp.
N-BEATS	0.25	11.2	0.743	0.756	0.712	**0.089**	**0.934**	Seq2Seq	0.25	12.1	0.761	0.741	0.694	**0.091**	**0.928**
	0.5	10.6	0.712	0.834	0.764	0.112	0.916		0.5	11.4	0.728	0.812	0.745	0.109	0.911
	0.75	10.1	0.689	0.876	0.798	0.128	0.897		0.75	10.9	0.703	0.863	0.789	0.129	0.892
	1.0	**9.8**	**0.672**	**0.924**	**0.847**	0.145	0.871		1.0	**10.6**	**0.694**	**0.907**	**0.832**	0.159	0.863
	1.25	9.9	0.678	0.918	0.841	0.156	0.863		1.25	10.7	0.699	0.901	0.826	0.171	0.855
	1.5	10.2	0.685	0.904	0.832	0.167	0.854		1.5	10.9	0.706	0.888	0.818	0.184	0.846
	1.75	10.5	0.694	0.893	0.824	0.178	0.845		1.75	11.2	0.714	0.876	0.809	0.197	0.837
	2.0	10.8	0.705	0.882	0.815	0.189	0.836		2.0	11.5	0.723	0.863	0.800	0.211	0.827
WaveNet	0.25	12.8	0.798	0.723	0.678	**0.095**	**0.921**	GRU	0.25	13.4	0.812	0.698	0.651	**0.099**	**0.915**
	0.5	12.1	0.764	0.792	0.726	0.115	0.903		0.5	12.7	0.778	0.769	0.703	0.118	0.898
	0.75	11.6	0.735	0.843	0.768	0.136	0.884		0.75	12.2	0.754	0.821	0.749	0.139	0.879
	1.0	**11.3**	**0.718**	**0.896**	**0.812**	0.168	0.834		1.0	**12.1**	**0.731**	**0.883**	**0.798**	0.172	0.847
	1.25	11.4	0.724	0.889	0.806	0.179	0.826		1.25	12.2	0.736	0.876	0.792	0.184	0.839
	1.5	11.7	0.731	0.876	0.798	0.191	0.817		1.5	12.4	0.743	0.863	0.784	0.197	0.830
	1.75	12.0	0.739	0.864	0.789	0.203	0.808		1.75	12.7	0.751	0.850	0.775	0.211	0.821
	2.0	12.3	0.748	0.851	0.780	0.216	0.799		2.0	13.0	0.760	0.836	0.765	0.225	0.811

Note: $\sigma_{frac}$ denotes the standard deviation fraction, representing the allowed change range relative to data standard deviation multiples when generating counterfactuals. Pred. Qual. = Prediction Quality; Val. = Validity; Prox. = Proximity; Comp. = Compactness. Lower values are better for sMAPE, MASE, and Proximity; higher values are better for Validity, Step-AUC, and Compactness. Bold values indicate optimal performance for each metric.

## Data Availability

The original data presented in the study are openly available on GitHub at https://github.com/xinxinluo123 (accessed on 3 December 2025).
